# Hurler syndrome or Morquio syndrome: Intelligence required for diagnosing the case

**DOI:** 10.4103/0971-3026.40306

**Published:** 2008-05

**Authors:** Amar Taksande, Krishna Vilhekar

**Affiliations:** Department of Pediatrics, Mahatma Gandhi Institute of Medical Sciences, Sevagram - 442 102, Wardha, Maharashtra, India. E-mail: amar_bharti2000@yahoo.co.uk

Dear Sir,

I have read with interest the article by Das *et al*.,[1] in which they have described a child with Hurler syndrome (MPS I), diagnosed on the basis of the clinical findings and radiological examination. In Hurler syndrome, the diagnosis is made between 6-24 months in the presence of hepatosplenomegaly, skeletal deformity, coarse facial features, corneal clouding, joint stiffness, and short stature. Hearing loss is common and developmental delay, with moderate mental retardation, is present.[2] Morquio syndrome, on the other hand, is characterized by short-trunk dwarfism, corneal deposits, skeletal dysplasia, and preservation of intelligence. Extraskeletal manifestations may include hepatomegaly, cardiac valvular lesions, and small teeth with caries formation.[2,3]

The author has not mentioned the status of mental development or the results of echocardiography.

In my opinion, this may be a patient with Morquio syndrome in view of the presence of hepatomegaly, cardiac valvular lesion, swollen alveolar ridges and gums with misaligned teeth, cloudy corneas, skeletal dysplasia, normal auditory functions, and normal intelligence.
